# Macrophage-derived pro-inflammatory cytokines augment the cytotoxicity of cytokine-induced killer cells by strengthening the NKG2D pathway in multiple myeloma

**DOI:** 10.1038/s41598-025-99289-x

**Published:** 2025-05-14

**Authors:** Peng Chen, Yinhao Chen, Yulu Wang, Amit Sharma, Lukacs-Kornek Veronika, Hans Weiher, A. Gonzalez-Carmona Maria, Ingo G. H. Schmidt-Wolf

**Affiliations:** 1 Department of Integrated Oncology, Center for Integrated Oncology (CIO), University Hospital Bonn, 53127 Bonn, Germany; 2 Jiangxi Provincial Key Laboratory of Hematological Diseases, Department of Hematology, The First Affiliated Hospital, Jiangxi Medical College, Nanchang University, Nanchang, China; 3 Department of Neurosurgery, University Hospital Bonn, 53127 Bonn, Germany; 4 Institute of Molecular Medicine & Experimental Immunology, University Hospital Bonn, 53127 Bonn, Germany; 5 Department of Applied Natural Sciences, Bonn-Rhein-Sieg University of Applied Sciences, 53359 Rheinbach, Germany; 6 Department of Internal Medicine I, University Hospital Bonn, 53127 Bonn, Germany

**Keywords:** Cytokine-induced killer cells, Multiple myeloma, Pro-inflammatory cytokines, Macrophages, MICA/B, PD-L1, Cancer, Immunology

## Abstract

Multiple myeloma (MM) is a clonal hematologic malignancy characterized by low rate of complete remissions. Cytokine-induced killer (CIK) cell therapy has shown promising benefits in MM treatment. In this study, we investigated whether the pro-inflammatory cytokines secreted by macrophages could upregulate MICA/B expression and thus the cytotoxicity of CIK cells. Flow cytometry was used for phenotypic measurement and the cytotoxicity assay of CIK cells. Soluble MICA/B and macrophage-derived cytokines were measured using ELISA assay. CCK-8 assay was applied to evaluate cell viabilities. Gene expression levels were investigated using RT-qPCR. The expression of MICA/B and PD-L1 in MM cells was upregulated by pro-inflammatory cytokines. Pro-inflammatory cytokines enhanced the cytotoxicity of CIK cells against MM cells, with TNF-α exhibiting a more potent effect than IL-1β and IL-6 as it strengthened both components of the NKG2D-MICA/B axis. PD-L1 blockade promoted the cytotoxic ability of CIK cells. Mechanistically, IL-1β, IL-6, and TNF-α enhanced the transcription of MICA/B and PD-L1 genes via the PI3K/AKT, JAK/STAT3, and MKK/p38 MAPK pathways. Pro-inflammatory cytokines upregulated the expression of MICA/B and PD-L1, thereby promoting the cytotoxicity of CIK cells against MM by strengthening the NKG2D pathway, while PD-L1 blockade enhanced the cytotoxicity of CIK cells.

**Supplementary Information:**

The online version contains supplementary material available at 10.1038/s41598-025-99289-x.

## Introduction

Multiple myeloma (MM) is a hematologic malignancy characterized by the proliferation of abnormal clonal plasma cells in the bone marrow and the overproduction of monoclonal immunoglobulins, which lead to destructive bone lesions, anemia, and kidney injury^1^. Among adoptive cellular immunotherapies (ACTs), cytokine-induced killer (CIK) cell therapy possesses specific advantages and has shown encouraging clinical efficacy in MM immunotherapy^2,3^. However, recurrence and treatment non-responsiveness remain critical issues, necessitating further in-depth research.

Derived from peripheral blood mononuclear cells (PBMCs), CIK cells are a heterogeneous population of exceptional T lymphocytes, characterized by the presence of CD3^+^CD56^+^ cells as their primary effectors. These cells possess both the functional properties of T cells and major histocompatibility complex (MHC)-unrestricted cytolysis, akin to NK cells^4,5^. Consequently, CIK cells have demonstrated significant efficacy in cancer immunotherapy, particularly against non-solid tumors^2^. Similar to NK cells, natural killer group 2D (NKG2D) plays a critical role in mediating the tumoricidal effects of CIK cells^6,7^. By engaging with its ligands, such as MHC class I chain-related protein A and B (MICA/B) and UL16 binding proteins (ULBPs)^8^, which are predominantly expressed on tumor cells and minimally on normal cells, NKG2D transmits activating signals to CIK cells. This activation induces polarization and the release of lytic granules, ultimately leading to the destruction of malignant cells.

Inflammation plays a pivotal role in tumor development and is a defining characteristic of the tumor microenvironment (TME)^9,10^. Among the immune cells within in the TME, macrophages are predominant and can be commonly classified into two subsets: classically activated (M1) and alternatively activated (M2) macrophages. M1 macrophages are characterized by the production of pro-inflammatory cytokines, including IL-1β, IL-6, and TNF-α^11^. Notably, pro-inflammatory cytokines have been reported to elicit dual roles in tumor biology, promoting either tumor inhibition or tumor progression depending on the context^12,13^.

In the present study, we aimed to investigate whether the pro-inflammatory cytokines influence the anti-tumor activity of CIK cells against MM cells. We demonstrated that macrophage-derived pro-inflammatory cytokines upregulated the expression of MICA/B and NKG2D in MM and CIK cells, respectively. In addition, these cytokines concurrently augmented programmed death-ligand 1 (PD-L1) expression in tumor cells. Notably, PD-L1 blockade effectively mitigated this immunosuppressive effect, restoring the anti-tumor activity of CIK cells.

## Results

### Pro-inflammatory cytokines upregulate the surface expression of MICA/B in MM cells

**Fig. 1:**
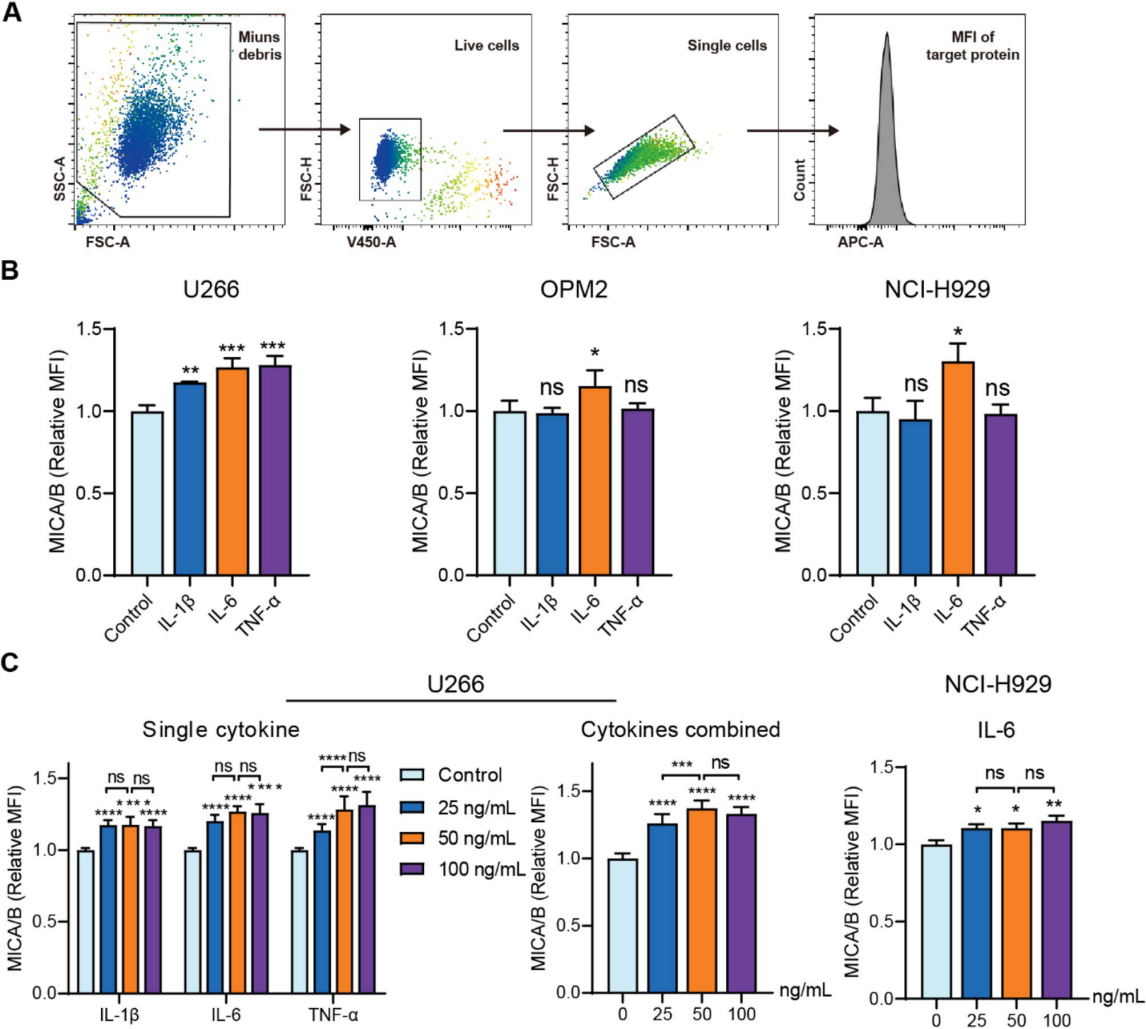
Pro-inflammatory cytokines upregulate MICA/B expression in MM. (**A**) Gating strategy for phenotype analysis. Debris and dead cells were sequentially excluded, and single cells were then gated for calculating the median fluorescence intensity (MFI) of targets. (**B**) Pro-inflammatory cytokines increased the surface expression of MICA/B in U266 cells, whereas only IL-6 acted on OPM2 and NCI-H929 cells. (**C**) In U266 cells, 50 ng/mL of single (left) or combined (right) cytokines exhibited the highest effect on MICA/B expression. In NCI-H929 cells, there was no significant difference among the IL-6 concentrations. Data are shown as mean ± SD of three independent experiments. *P < 0.05, **P < 0.01, ***P < 0.001, ****P < 0.0001, ns, not significant, calculated by one-way (**B**, **C**) or two-way (**C**) ANOVA, Bonferroni’s post-hoc test.

To investigate whether pro-inflammatory cytokines influence MICA/B expression in MM cells, we first incubated three MM cell lines (U266, OPM2 and NCI-H929) with 50 ng/mL of cytokines and measured surface MICA/B expression. The gating strategy was shown in Fig. 1A. The results showed that U266 cells were sensitive to all three cytokines, whereas OPM2 and NCI-H929 cells responded only to IL-6 (Fig. 1B). To optimize cytokine concentrations, U266 and NCI-H929 cells were treated with a gradient of cytokine concentrations. For U266 cells, 50 ng/mL of TNF-α exhibited significantly higher MICA/B expression than 25 ng/mL or 100ng/mL, whereas IL-1β and IL-6 showed no significant differences across concentrations (Fig. 1C). For NCI-H929 cells, IL-6 elicited comparable effects at all tested concentrations (Fig. 1C). In addition, when U266 cells were treated with combined cytokines, 50 ng/mL proved more effective than 25 ng/mL but showed no significant difference compared to 100 ng/mL (Fig. 1C). For convenience and consistency, 50 ng/mL was chosen as the working concentration for subsequent experiments.

### Pro-inflammatory cytokines enhance CIK cell-mediated cytotoxicity

**Fig. 2:**
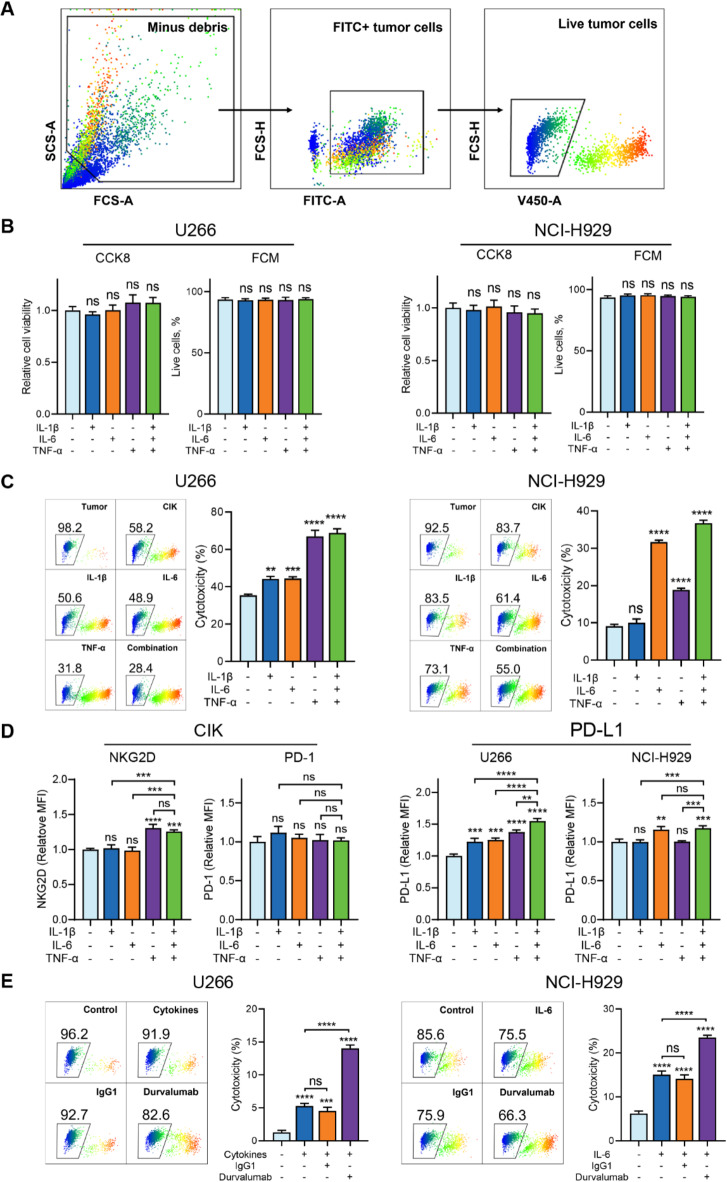
Pro-inflammatory cytokines promote the cytotoxicity of CIK cells against MM. (**A**) Gating strategy for cytotoxicity analysis. Debris were excluded and FITC + tumor cells were then gated to exclude CIK cells (FITC-) in co-culture system. Live tumor cells were gated and analyzed. (**B**) Potential cytotoxic effects of cytokines on tumor cells were excluded by FCM and CCK-8 assays. (**C**) In U266 cells, all cytokines increased the cytotoxicity of CIK cells, whereas in NCI-H929 cells, only IL-6 and TNF-α enhanced the cytotoxic activity of CIK cells, but not IL-1β. (**D**) TNF-α upregulated NKG2D expression in tumor cells, whereas none of the cytokines affected PD-1 expression in CIK cells. All cytokines augmented PD-L1 expression in U266 cells, while only IL-6 acted on NCI-H929 cells. (**E**) PD-L1 blockade enhanced the CIK cells’ cytotoxicity against MM cells. Durvalumab, PD-L1 antibody, 10 µg/mL. Data are shown as mean ± SD of three independent experiments. *P < 0.05, **P < 0.01, ***P < 0.001, ****P < 0.0001, ns, not significant, calculated by one-way ANOVA, Bonferroni’s post-hoc test.

To determine whether pro-inflammatory cytokines enhance the cytotoxicity of CIK cells against MM cells via modulating the expression of MICA/B, we first assessed the potential direct cytotoxic effects of cytokines. The gating strategy for cytotoxicity analysis was shown in Fig. 2A. Cell viability and live cell percentages were measured and confirmed that the cytokines themselves had no cytotoxicity on MM cells (Fig. 2B). Subsequently, we co-incubated cytokine-treated MM cells with CIK cells, and observed that cytokine treatment significantly enhanced the cytotoxicity of CIK cells against MM cells (Fig. 2C). Interestingly, in U266 cells, TNF-α treatment significantly enhanced the cytotoxicity of CIK cells compared to IL-1β and IL-6, although their similar effects on MICA/B modulation (Figs. 1C and 2C). In NCI-H929 cells, TNF-α also promoted CIK cell-mediated killing, despite having no effect on MICA/B expression (Figs. 1C and 2C). These findings suggest that TNF-α exerts a unique and potent effect on boosting the cytotoxicity of CIK cells, likely via mechanisms beyond MICA/B modulation.

NKG2D is a key mediator of CIK cell cytotoxicity^6,7^. Our data demonstrate that inhibiting NKG2D in CIK cells significantly reduces their killing activity (Supplementary Fig. 1). To explore this hypothesis, we examined the expression of NKG2D, the receptor for MICA/B, on CIK cells following cytokine treatment. Results demonstrated that TNF-α, but not IL-1β and IL-6, significantly increased NKG2D expression on CIK cells (Fig. 2D). Meanwhile, we assessed PD-1 and PD-L1 expression on CIK cells and MM cells. Interestingly, all cytokines elevated PD-L1 expression in U266 cells, whereas only IL-6 augmented PD-L1 expression in NCI-H929 cells (Fig. 2D). However, none of the cytokines influenced PD-1 expression on CIK cells (Fig. 2D). Given the upregulated PD-L1 on MM cells, we further investigated whether PD-L1 blockade could enhance CIK cell-mediated cytotoxicity. As anticipated, treatment with a PD-L1 antibody significantly increased the cytotoxicity of CIK cells against both U266 and NCI-H929 cells compared to cytokine treatment alone (Fig. 2E).

Taken together, these data indicate that pro-inflammatory cytokines enhance the cytotoxicity of CIK cells, with TNF-α showing a particularly pronounced effect compared to IL-1β and IL-6, as it simultaneously reinforces both components of the NKG2D pathway. In addition, these cytokines also elevate PD-L1 expression, while PD-L1 blockade significantly restores and enhances the cytotoxic potential of CIK cells.

### IL-1β, IL-6 and TNF-α promote the transcription of MICA/B and PD-L1 gene via PI3K/AKT, JAK/STAT3, and MKK/p38 MAPK pathways

**Fig. 3:**
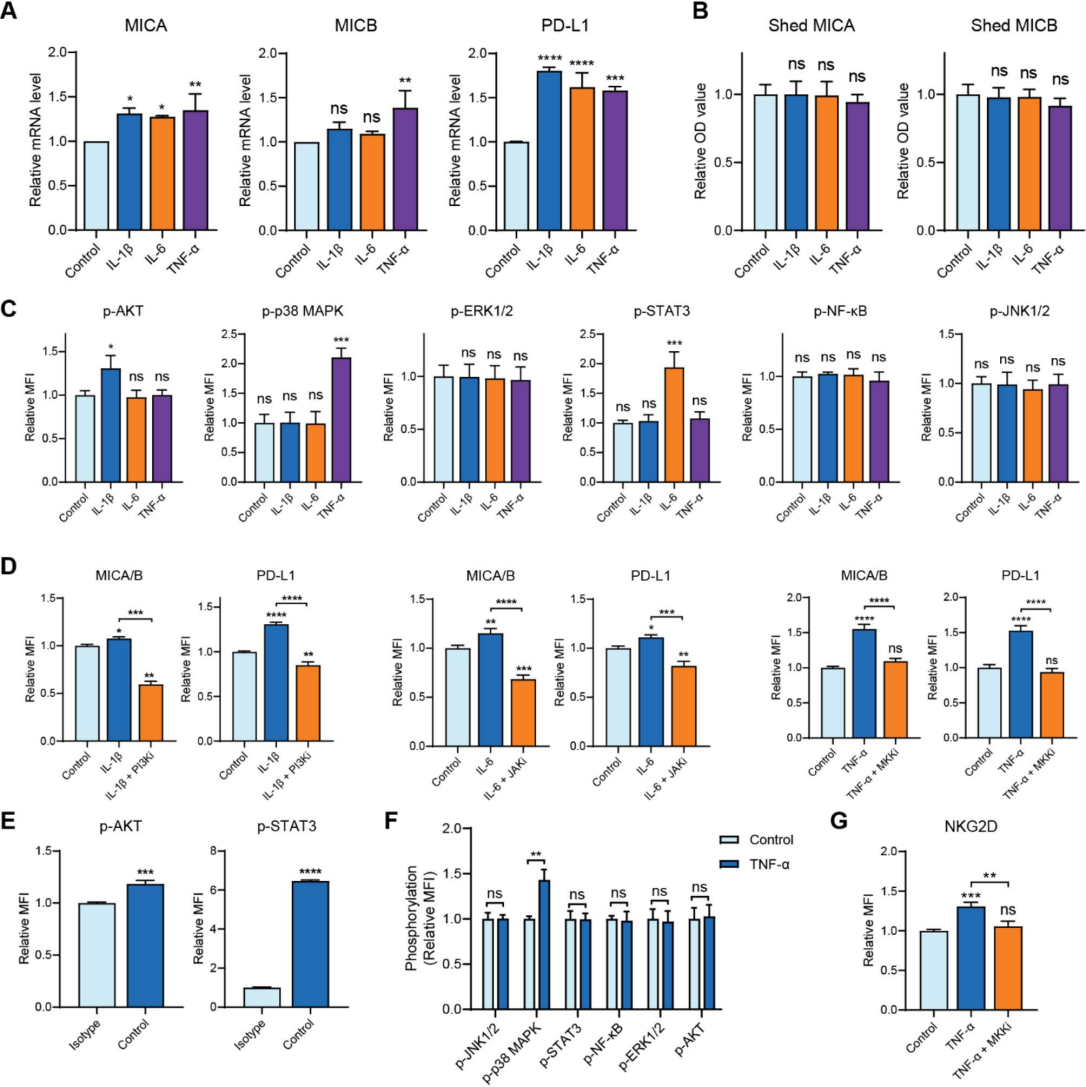
Pro-inflammatory cytokines promote the transcription of MICA/B and PD-L1 genes in U266 cells. (**A**) Pro-inflammatory cytokines enhanced the transcription of MICA and PD-L1 genes while only TNF-α upregulated MICB transcription. (**B**) Cytokine treatments did not affect the levels of shed MICA and MICB. (**C**) The phosphorylation levels of AKT, p38 MAPK, and STAT3 increased upon the treatment of IL-1β, TNF-α, and IL-6, respectively, while ERK1/2, NF-κB, and JNK remained unchanged. (**D**) Inhibitors of PI3K, JAK, and MAPK completely reversed the upregulation of MICA/B and PD-L1 caused by cytokines. PI3Ki, LY-294,002, 10 µM; MMKi, Gossypetin, 60 µM; JAKi, JAK inhibitor I, 1 µM. (**E**) STAT3 and AKT were constitutively activated in tumor cells. (**F**) TNF-α activated p38 MAPK in CIK cells. (**G**) Inhibition of MKK abolished TNF-α-induced upregulation of NKG2D in CIK cells. Data are shown as mean ± SD of three independent experiments. *P < 0.05, **P < 0.01, ***P < 0.001, ****P < 0.0001, ns, not significant, calculated by one-way ANOVA, Bonferroni’s post-hoc test (**A**–**D**, and **G**) or student’s unpaired t test (**E**, **F**).

To identify the mechanisms by which pro-inflammatory cytokines modulate MICA/B and PD-L1, we first evaluated the transcription levels of MICA, MICB, and PD-L1. The results revealed that cytokine treatments promoted the mRNA levels of MICA and PD-L1, while only TNF-α upregulated MICB mRNA levels (Fig. 3A). ELISA assays were then performed to assess shed MICA and MICB after cytokine treatment. The results showed that the levels of shed MICA and MICB remained comparable to those in the control group, suggesting that the elevated expression of MICA/B was not attributable to altered shedding (Fig. 3B).

To elucidate the specific signaling pathways activated by cytokines, we examined several pathways commonly associated with cytokine signaling, including Janus kinase (JAK)/signal transducer and activator of transcription (STAT), nuclear factor-κB (NF-κB), mitogen-activated protein kinase (MAPK), and phosphatidylinositol 3‑kinase (PI3K)/protein kinase B (AKT). Upon cytokine treatment, we observed increased phosphorylation levels of AKT, STAT3, and p38 MAPK, while c-Jun NH2-terminal kinase 1/2 (JNK) 1/2, extracellular signal-regulated kinase 1/2 (ERK1/2), and NF-κB remained unchanged (Fig. 3C). To further identify the involvement of these pathways, we evaluated MICA/B and PD-L1 expression in the presence of specific inhibitors targeting JAK, PI3K, and mitogen-activated protein kinase kinase (MKK). The upregulation of MICA/B and PD-L1 induced by cytokines was completely abolished when inhibitors were applied (Fig. 3D). Interestingly, the inhibition of JAK and PI3K reduced MICA/B and PD-L1 expression to levels even lower than those in the control groups (Fig. 3E), suggesting constitutive activation of the JAK/STAT3 and PI3K/AKT pathways in MM. Consistent with this observation, basal levels of STAT3 and AKT phosphorylation were notably high in the absence of cytokine stimulation (Fig. 3E).

Given that TNF-α upregulated NKG2D expression in CIK cells, we next explored the underlying signaling pathways in CIK cells. The results demonstrated that TNF-α also activated the p38 MAPK pathway in CIK cells (Fig. 3F). Furthermore, TNF-α-induced upregulation of NKG2D in CIK cells was abolished by inhibiting this pathway (Fig. 3G). Overall, our findings reveal that pro-inflammatory cytokines elevated MICA/B and PD-L1 expression via transcription, and identify the signaling pathways through which cytokines function.

### M1 macrophage-derived pro-inflammatory cytokines increase MICA/B and PD-L1 expression and the cytotoxicity of CIK cells against MM

Considering that M1 macrophages are the primary source of pro-inflammatory cytokines in the TME^14^, we induced M1 macrophages from THP-1 cells and investigated whether the cytokines secreted from M1 macrophages exert comparable effects to those of human recombinant cytokines. The polarization of M1 macrophages was initially identified based on their morphological features and the CD86/CD206 expression patterns. Morphologically, M1 macrophages displayed a mixture of dendritic-like and spindle-shaped cells and were predominantly CD86^+^CD206^−^ (Supplementary Fig. 2A and B). The secretion of pro-inflammatory cytokines was subsequently confirmed using ELISA assays. The results showed that IL-1β, IL-6, and TNF-α were highly secreted by M1 macrophages (Supplementary Fig. 2C).

**Fig. 4:**
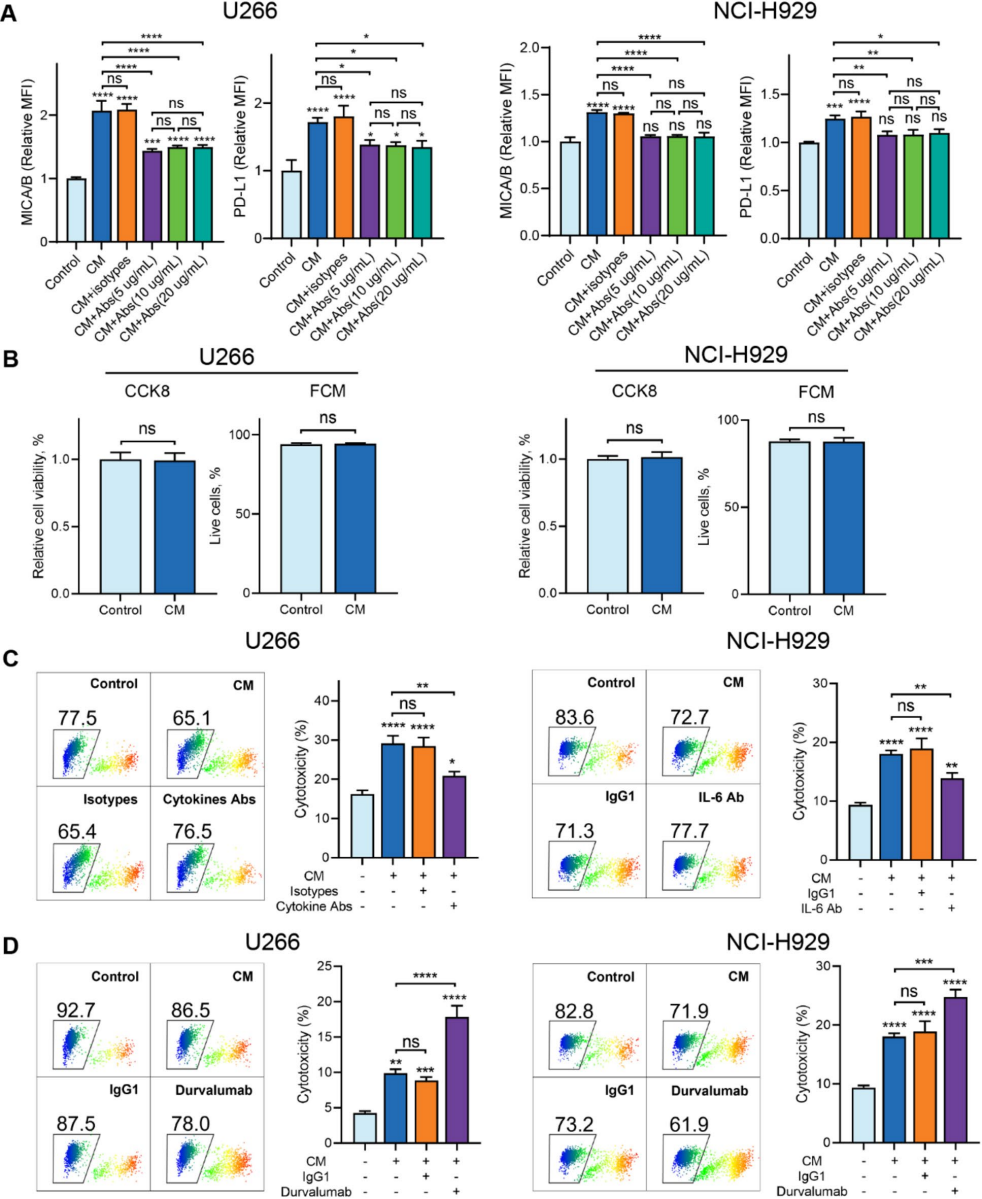
Macrophage-derived pro-inflammatory cytokines upregulate the expression of MICA/B and the cytotoxicity of CIK cells. (**A**) CM from M1 macrophages upregulated MICA/B and PD-L1 expression in tumor cells, and the cytokine antibodies partially or completely eliminated this effect. (**B**) CM had no cytotoxic effect on tumor cells. (**C**) CM promoted the cytotoxicity of CIK cells against MM cells, while the use of cytokine antibodies (anti-IL-1β, anti-IL-6, and anti-TNF-α antibodies for U266 cells, anti-IL-6 antibody for NCI-H929 cells) partially abolished the cytotoxicity of CIK cells. (**D**) Blockade of PD-L1 enhanced the cytotoxicity of CIK cells. Durvalumab, PD-L1 antibody, 10 µg/mL. Data are shown as mean ± SD of three independent experiments. *P < 0.05, **P < 0.01, ***P < 0.001, ****P < 0.0001, ns, not significant, calculated by one-way ANOVA, Bonferroni’s post-hoc test (**A**, **C**, **D**) or student’s unpaired t test (**B**).

For MICA/B and PD-L1 measurement, tumor cells were co-cultured with CM from M1 macrophages, with or without anti-IL-1β, anti-IL-6, and anti-TNF-α antibodies. The results showed that CM significantly upregulated MICA/B and PD-L1 expression, while cytokine antibodies partially or completely reversed this effect (Fig. 4A). For cytotoxicity assessment, we first confirmed that CM itself exhibited no direct cytotoxicity against tumor cells (Fig. 4B). Co-culture of tumor cells with CM enhanced the cytotoxicity of CIK cells, whereas cytokine antibodies partially reversed this enhanced cytotoxicity (Fig. 4C). Notably, PD-L1 blockade further augmented the cytotoxic activity of CIK cells against MM (Fig. 4D).

In summary, those results demonstrate that macrophage-derived pro-inflammatory cytokines upregulate MICA/B and PD-L1 expression, enhancing the cytotoxicity of CIK cells against MM. Additionally, PD-L1 blockade contributes to the cytotoxicity of CIK cells against MM.

## Discussion

The crosstalk between inflammation and tumor cells in the TME is highly intricate and multifaced. Inflammation within the TME is widely recognized to promote tumorigenesis by interfering with anti-tumor immunity, reshaping the TME towards a tumor-supportive niche, and acting as tumor-promoting signals for epithelial and cancer cells^10^. However, the specific role of pro-inflammatory cytokines in the TME appears to be more nuanced, as they often exhibit dual and seemingly opposing effects in tumor immunity. As demonstrated in this study, macrophage-derived pro-inflammatory cytokines enhanced the anti-tumor activity of CIK cells against MM by boosting the NKG2D/NKG2D ligand axis. Concurrently, these cytokines also facilitated immune evasion by augmenting PD-L1 expression on tumor cells. Previous studies have confirmed that, in addition to macrophages, other cellular components within the TME, including tumor cells, can also produce substantial amounts of pro-inflammatory cytokines and suppress anti-tumor immunity through mechanisms such as PD-L1 upregulation^10,15,16^.

Unlike pro-inflammatory cytokines, M1 macrophages, the major producer of pro-inflammatory cytokines in the TME, are generally considered supportive of anti-tumor immunity^14,17^. However, “educated” by environmental signals in the TME, macrophages can acquire a M2-like phenotype and transform into tumor-associated macrophages (TAMs), which are known to facilitate tumor progression^18–20^. Due to their high plasticity, M1 and M2 macrophages are interconvertible in response to changes in the TME or therapeutic interventions^18^. Reprogramming macrophages to shift M2 macrophages toward M1 phenotype has emerged as a promising strategy to counteract the immunosuppression status of the TME. Various approaches, ranging from conventional targeted antibodies and small molecule drugs to advanced gene modification technologies, such as nucleic acids and viral vectors have been developed for macrophage reprogramming and have shown promising clinical benefits^21^. In recent years, significant advances have been made in TAM-targeted reprogramming therapies. Small molecule inhibitors, such as CSF1R and PI3K-γ inhibitors, can reduce M2-like TAM recruitment and immunosuppressive activity^22,23^. Immune checkpoint modulation, including anti-CD47 antibodies, enhances macrophage-mediated phagocytosis^24^. TLR agonists promote M1-like activation^24^. Nanotechnology enables targeted drug delivery for M2-to-M1 repolarization^25^. In addition, metabolic reprogramming is another promising strategy. Inhibiting glucose metabolism, lipid metabolism, and arginine metabolism can suppress M2-like TAM activation and proliferation^26^.

However, our results indicated that pro-inflammatory cytokines secreted by M1 macrophages paradoxically increase PD-L1 expression on tumor cells. Additionally, studies have revealed that PD-L1 expression on macrophages also increases following reprogramming therapies, thereby contributing to immune evasion^27^. Consequently, we propose that including PD-L1 blockade within macrophage reprogramming therapies could effectively mitigate PD-L1-mediated immunosuppression while enhancing the anti-tumor efficacy.

Our findings demonstrated that pro-inflammatory cytokines utilize the JAK/STAT3, MAPK, and PI3K/AKT signaling pathways to modulate MICA/B expression. Notably, these pathways are widely considered as tumor-favoring signals that drive tumorigenesis and progression, with aberrant hyperactivation observed in the majority of human malignancies and correlated with poor clinical outcomes^28–31^. Consequently, the development of targeted inhibitors for these pathways has become a critical approach of anti-tumor therapies, yielding promising clinical results^30–32^. However, our data highlighted a paradox: inhibiting these pathways may inadvertently suppress MICA/B expression, thereby impairing innate immune responses against tumors. Regarding PD-L1, these pathways have been revealed to contribute to augmented PD-L1 in tumors^33–35^. In this context, inhibitors targeting these pathways may also act as PD-L1 suppressants. Nevertheless, we recommend prioritizing the use of PD-L1 antibodies over signaling inhibitors or cytokine-targeted therapies, as the latter could downregulate MICA/B, potentially compromising anti-tumor immunity.

Moreover, while CIK cells share functional similarities with NK cells, their responses to pro-inflammatory cytokines are notably distinct. Our results found that IL-6 significantly increased the cytotoxicity of CIK cells, however, previous studies demonstrated that tumor-derived IL-6 and IL-8 impair the anti-tumor activity of NK cells^36,37^. We suggest that this divergence highlight a potential advantage of CIK cells over NK cells in tumor immunotherapy. However, the underlying mechanisms remain unclear and warrant further investigation to fully elucidate the unique properties of CIK cells.

**Fig. 5:**
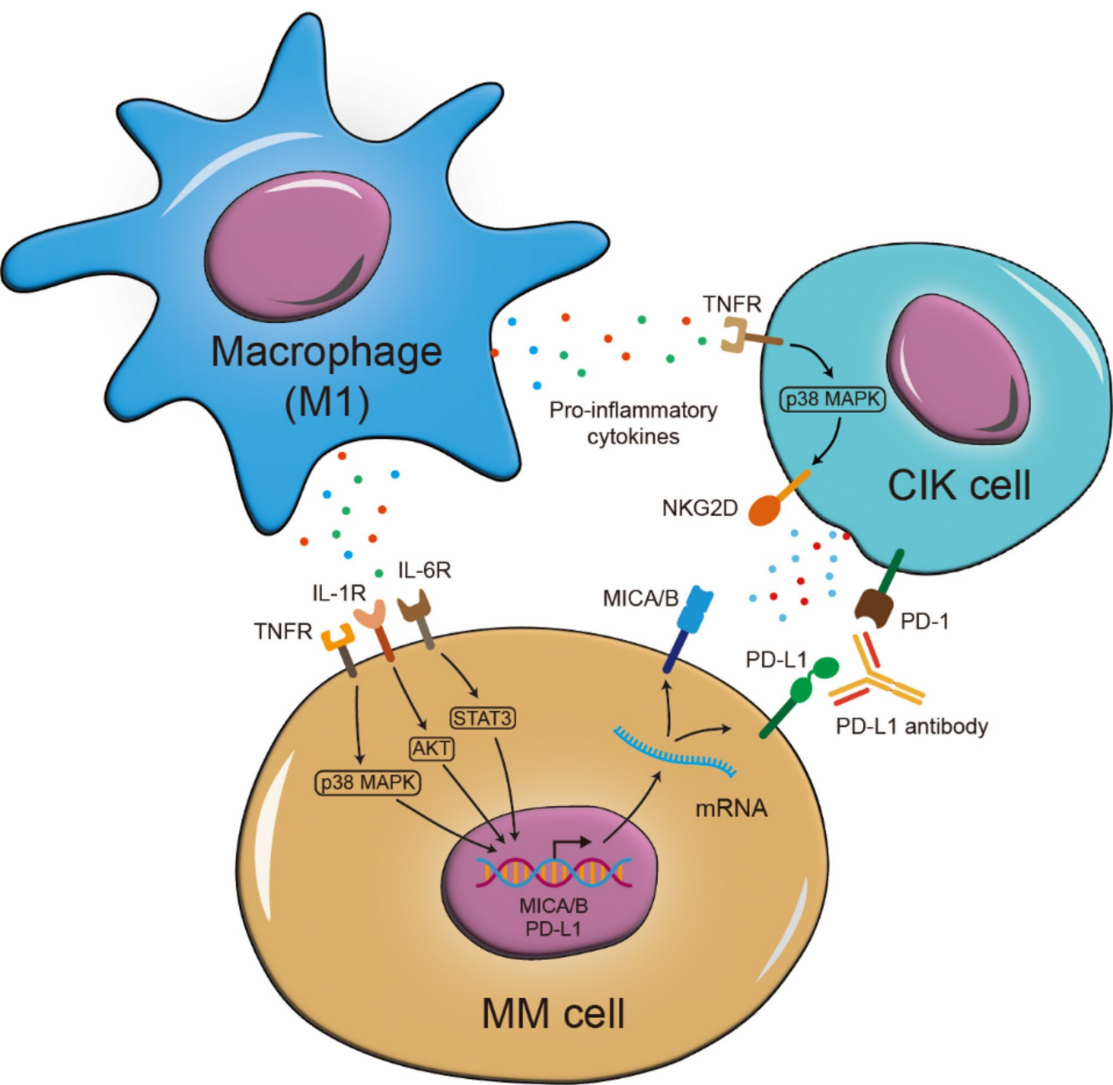
Illustrative diagram of macrophage-derived pro-inflammatory cytokines enhance the cytotoxicity of CIK cells. Macrophage-derived pro-inflammatory cytokines (IL-1β, IL-6, and TNF-α) target both tumor cells and CIK cells to strengthen the NKG2D-MICA/B axis and thus promoting the cytotoxicity of CIK cells against MM. In addition, pro-inflammatory cytokines also upregulate PD-L1 expression in tumor cells, and PD-L1 blockade therapy can restore this immunosuppression and enhance the cytotoxicity of CIK cells.

We observed that M1 macrophage-derived pro-inflammatory cytokines simultaneously activated the NKG2D and PD-L1 pathway, suggesting that macrophage reprogramming may favorably affect tumors via PD-L1 (Fig. 5). However, this study utilized only M1 macrophages under in vitro conditions, further in-depth in vivo and in vitro studies are required to validate these observations by using macrophage reprogramming approaches, such as small molecules drugs or gene editing techniques.

In conclusion, we demonstrated that macrophage-derived pro-inflammatory cytokines promote MICA/B and PD-L1 expression in MM by upregulating gene transcription. The enhanced NKG2D-MICA/B axis boosts CIK cell-mediated cytotoxicity, while PD-L1 blockade mitigates the immunosuppression induced by upregulated PD-L1 and further enhances the cytotoxicity of CIK cells. Our findings reveal a novel mechanism by which pro-inflammatory cytokines enhance anti-tumor immunity and propose a combination treatment with PD-L1 blockade during macrophage reprogramming therapy.

## Methods and materials

### Cell culture and CIK cells generation

Human MM cell lines U266, OPM2, and NCI-H929, and human monocyte cell line THP-1 were purchased from the American Type Culture Collection (ATCC; Manassas, VA, USA) and were maintained in RPMI 1640 medium (Pan-Biotech, Aidenbach, Germany) supplemented with 10% FBS (Gibco, Waltham, MA, USA) and 1% penicillin–streptomycin (Gibco) at 37 °C, 5% CO_2_. Mycoplasma contamination was tested negatively prior to the use. CIK cells were generated and cultured as previously described^7^.

### Antibodies, inhibitors and cytokines

Antibodies used in flow cytometry were purchased from BioLegend (Koblenz, Germany), including APC anti-MICA/B (6D4), PE anti-PD-L1 (MIH2), APC anti-CD86 (BU63), PE anti-CD206 (15 − 2), anti-CD16/32 (93), APC anti-PD-1 (EH12.2H7), FITC anti-NKG2D (1D11), PE anti-p38 MAPK Phospho (A16016A), PE anti-STAT3 Phospho (13A31), APC anti-NF-κB Phospho (14G10A21), PE anti-ERK1/2 Phospho (6B8B69), APC Mouse IgG2a, κ (MOPC-173), PE Mouse IgG1, κ (MOPC-21), APC Mouse IgG1, κ (MOPC-21), and PE Rat IgG2a, κ (RTK2758). Phosflow PE anti-JNK1/2 (N9-66) and Phosflow PE-AKT (M89-61) were purchased from BD Bioscience (San Diego, CA, USA). Human cytokine neutralizing antibodies and matched isotypes were purchased from Invitrogen (Carlsbad, CA, USA), including anti-TNF-α (MAb1), anti-IL6 (MQ2-13A5), anti-IL1β (CRM56), Mouse IgG1, κ (P3.6.2.8.1), and Rat IgG1, κ (eBRG1). JAK inhibitor (JAK inhibitor I, #420097) and PI3K inhibitor (LY-294002, #L9908) were purchased from Merck (Darmstadt, Germany). MKK3/6 inhibitor (Gossypetin, #Cay33840) was purchased from Biomol (Hamburg, Germany). Recombinant human IL-1β (#11340015), IL-2 (#11340025), IL-6(#11340064), TNF-α (#11343015), and IFN-γ (#11343534) were from ImmunoTolls (Aidenbach, Bavaria, Germany). PD-L1 antibody (Durvalumab, #A2013) was purchased from Selleckchem (Houston, TX, USA).

### Flow cytometry

Flow cytometry (FCM) were performed on a FACSCanto II (BD Bioscience) machine. For phenotype analysis, cells were seeded with cytokines or conditioned-medium (CM) from M1 macrophages for 24 h. Then, cells were collected and washed once followed by antibody staining at 4 °C for 30 min. Next, cells were washed twice and resuspended in 200 µl DPBS for analysis. Hoechst 333,258 (Cayman Chemical, Hamburg, Germany) was used to recognize dead cells. Of note, for Fc receptors blockade, macrophages were incubated with CD16/32 antibodies at 4 °C for 10 min prior to the antibody staining. For the inhibition of signaling pathways, cells were pre-treated with the inhibitors for 20 min prior to the treatment of cytokines. CM was pre-incubated with cytokine antibodies at 4 °C for 3 h for cytokines neutralization before the use.

For cytotoxicity assays, 1 × 10^4^ of CFSE-labeled tumor cells were seeded with cytokines or CM for 24 h. CIK cells were then added to tumor cells and co-cultured for 24 h followed by analysis. For the neutralizing and blocking experiments, prior to the co-culturing with CIK cells, cytokine antibodies or PD-L1 antibody were pre-incubated with tumor cells at 4°C for 3 h or at 37 °C for 2 h, respectively. The formula used for cytotoxicity calculation is as follows: Cytotoxicity = CL-TL. CL, live tumor cells percentage in tumor cells alone group; TL, live tumor cells percentage in tumor cells and CIK cells groups, with or without treatments. Effector to target (ET) ratio was 10:1 in this study.

For intracellular staining, cells were pre-incubated with cytokines for 30 min, and cells were then stained with Zombie Aqua Dye (BioLegend) for 15 min to distinguish dead cells. Next, cells were fixed and permeated with IC Fixation Buffer (Thermo Scientific, Waltham, MA, USA) and cold methanol. Finally, cells were incubated with phospho-antibodies followed by analysis.

### Gene expression analysis

Total RNA was isolated with RNeasy Plus Mini Kit (QIAGEN, Hilden, Germany). The quality of RNA samples was measured using Nanodrop1000 (NanoDrop, Wilmington, DE, USA). The complementary DNA (cDNA) was synthesized with High-Capacity cDNA Reverse Transcription Kit (Applied Biosystems, Foster City, CA, USA). Gene quantification was performed on a QuantStudio3 system (Applied Biosystems) using TaqMan Gene Expression Assay (Applied Biosystems). Gene expression levels were normalized to GAPDH. 2^−ΔΔCt^ method was used to calculate relative expression. Pre-designed TaqMan primers GAPDH (Hs99999905_m1), MICA (Hs07292198_gH), MICB (Hs00792952_m1), and PD-L1 (Hs00204257_m1) were purchased from Applied Biosystems.

### Cell counting kit-8 (CCK-8) assay

5 × 10^4^ U266 or NCI-H929 cells well seeded with cytokines or CM. After 24 h, 10ul of CCK-8 solution (Dojindo, Kumamoto, Japan) was added to wells and incubated for 1 h (U266) or 3 h (NCI-H929). The absorbance (OD value) at 450 nm was measured on Multiskan GO Microplate Spectrophotometer (Thermo Scientific). The viability of tumor cells was calculated using the following formula: Viability = (OD experimental - OD blank) / (OD control − OD blank) × 100%.

### ELISA assay

Supernatants from tumor cells and M1 macrophages were collected for the measurement of shed MICA/B and cytokines, respectively. For cytokines measurement, supernatants were 10 folds diluted prior to the use. The shed MICA (#DY1300) and MICB (#DY1599) ELISA kits were purchased from R&D System (Minneapolis, MN, USA), and TNF-α (#88-7346-22), IL-6 (#88-7066-22) and IL-1β (#88-7261-22) ELISA kits were purchased from Invitrogen. ELISA assays were conducted according to the manufacturer’s instructions.

### Macrophages polarization and characterization

The protocol of macrophages polarization from THP-1 cells has been described previously, with our minor modifications^38^. Briefly, 5 × 10^5 THP-1 cells were seeded to 6-well plates and stimulated to M0 macrophages by adding 100 ng/mL phorbol 12-myristate 13-acetate (PMA; #P8139, Sigma-Aldrich, St. Louis, MO, USA) for 24 h. For the polarization of M1 macrophages, M0 macrophages were then treated with 100 ng/mL LPS (#L4391, Sigma-Aldrich) and 20 ng/mL IFN-γ for 48 h in the presence of 50 ng/mL PMA.

### Statistical analysis

Flow cytometry data sets were analyzed using FlowJo v10.6 software (Tree Star, Ashland, OR, USA). Statistical analyzes were performed using GraphPad Prism v.8.0 (GraphPad Software, La Jolla, CA, USA). Quantitative data were presented as mean ± SD (standard deviation). Differences between groups were investigated using Student’s unpaired *t* test and one- or two-way analysis of variance (ANOVA) with Bonferroni’s *post-hoc* test. Each experiment was performed for three times. P *≤* 0.05 was considered statistically significant.

## Electronic supplementary material

Below is the link to the electronic supplementary material.

Supplementary Material 1

Supplementary Material 2

## Data Availability

The datasets generated during the current study are available from the corresponding author on reasonable request.
