# Supplementary figures and images for: Macrophage-derived pro-inflammatory cytokines augment the cytotoxicity of cytokine-induced killer cells by strengthening the NKG2D pathway in multiple myeloma
Source: Sci Rep. 2025 May 14;15:16739. doi: 10.1038/s41598-025-99289-x (PMC12078699; doi:10.1038/s41598-025-99289-x)

## Supplementary Figure 1

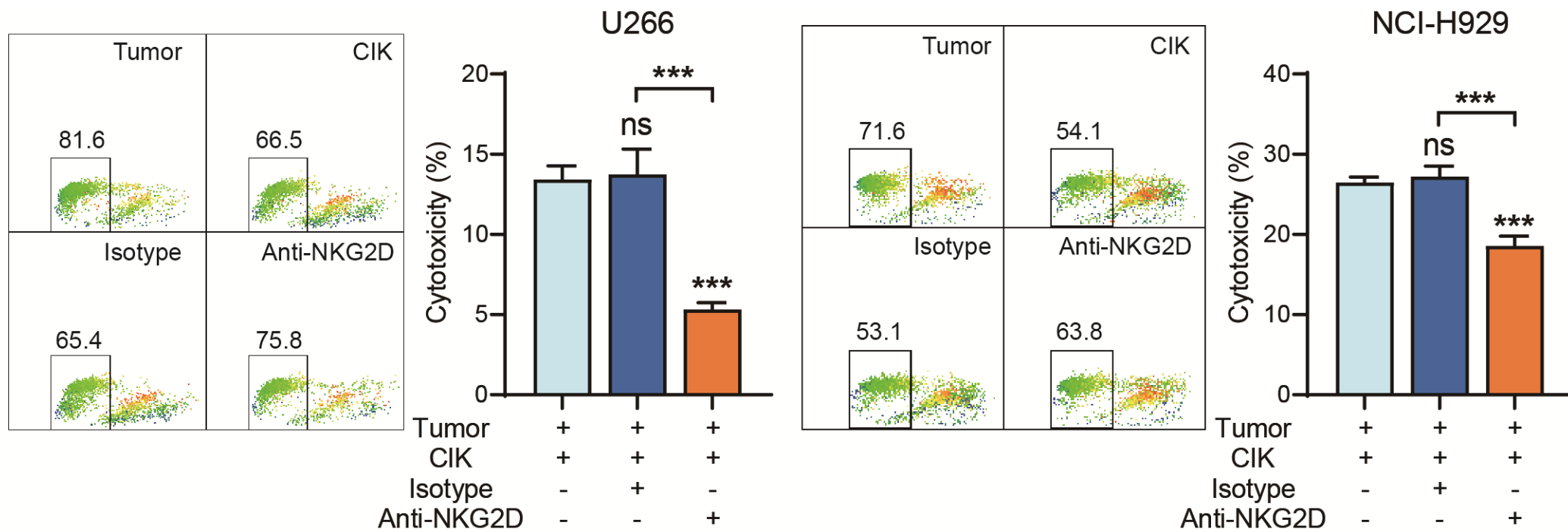

Supplementary Figure 2

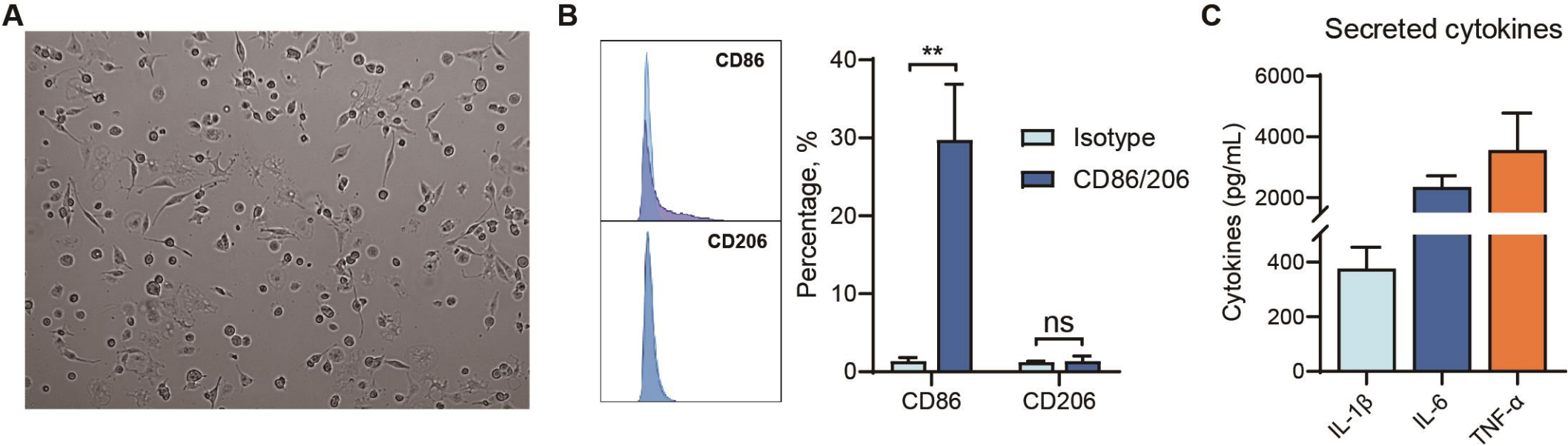

Supplement: Supplementary file 1 — Supplementary Material 1 [file 41598_2025_99289_MOESM1_ESM.pdf]
