# Supplementary material for: Macrophage-derived pro-inflammatory cytokines augment the cytotoxicity of cytokine-induced killer cells by strengthening the NKG2D pathway in multiple myeloma
Source: Sci Rep. 2025 May 14;15:16739. doi: 10.1038/s41598-025-99289-x (PMC12078699; doi:10.1038/s41598-025-99289-x)
Supplement: Supplementary file 2 — Supplementary Material 2 [file 41598_2025_99289_MOESM2_ESM.docx]

Supplementary Figure Legends

Supplemental Figure 1. Blockade of NKG2D impairs the cytotoxicity of CIK cells. Inhibition of the NKG2D on CIK cells using NKG2D antibody significantly suppressed the killing activity of CIK cells against MM cells. Data are shown as mean ± SD of three independent experiments. ***P < 0.001, ns, not significant, calculated by one-way ANOVA, Bonferroni’s post-hoc test.

Supplemental Figure 2. The polarization of M1 macrophages. (A) Representative brightfield microscope image of morphological features of M1 macrophages. (B) M1 macrophages expressed CD86 but not CD206. (C) M1 macrophages highly secreted IL-1β, IL-6, and TNF-α. Data are shown as mean ± SD of three independent experiments (B, C) or one representative of three independent experiments (A). **P < 0.01, ns, not significant, calculated by student’s unpaired t test (B).
